# How Do Ecological and Recreational Features of Waterfront Space Affect Its Vitality? Developing Coupling Coordination and Enhancing Waterfront Vitality

**DOI:** 10.3390/ijerph20021196

**Published:** 2023-01-10

**Authors:** Lihua Chen, Yuan Ma

**Affiliations:** School of Architecture and Urban Planning, Landscape Planning and Ecological Restoration Research Center, Guangdong University of Technology, Guangzhou 510090, China

**Keywords:** waterfront space, ecological-recreational coupling, natural environment quality, recreational service capacity, spatial vitality

## Abstract

People are increasingly concerned with natural environment quality (NEQ) as well as recreation services (RS) as the popularity of natural experiences grows. Specifically, public spaces in ecologically sensitive areas must achieve coordinated eco-recreational development. Waterfront spaces fall into this category, providing a high-quality natural environment and facilitating various recreational activities. This paper uses two waterfront spaces, Foshan New City Riverfront Park and Nanhai Qiandeng Lake Park, as sample sites, divides 22 waterfront space samples into specific research objects, introduces dual variables for RS function and NEQ, and uses mathematical and statistical methods, such as Pearson correlation analysis, coupling coordination degree calculation, and redundancy analysis, to investigate the influence of different waterfront spaces on RS function and NEQ. Finally, we propose an optimization strategy for coupling and coordinating the development of the RS function and the NEQ of waterfront space. This paper found the following: (1) RS (number of public facilities) and natural environment quality (shoreline curvature) are the dominant factors in the vitality of waterfront space; (2) optimization of RS function will restrict the development of NEQ to a certain extent; and (3) the coupling and coordination of NEQ and RS function are positively related to the vitality of waterfront space. This study can be valuable for government officials and waterfront space planners as they develop social–ecological synergy models.

## 1. Introduction

It is evident that waterfront renewal and regeneration is one of the world’s most highly valued initiatives, and it has gained support from both the public and government officials over the past few years [1]. It is widely accepted that cities use riverfronts to create various open spaces. These open spaces are typical blue–green spaces and are the best way to connect urban dwellers with nature [2], providing various services and benefits, including stress relief, exercise, social interaction, and recreational activities for the general population. Residents may regularly engage in spontaneous and passive activities when such waterfront spaces are present. Therefore, it is believed that waterfront spaces contribute to their well-being and quality of life and that the waterfront space positively affects their lives [3]. Due to the rise of leisure and natural experiences, residents’ recreational behavior has gradually shifted from open spaces in urban centers to the suburbs. In recent years, the waterfront has become significantly more appealing to residents. In a region with more natural resources, a natural space can provide a greater variety of recreation services than a city center to meet the public’s diverse leisure needs. Due to the COVID-19 pandemic, there has been a substantial increase in the number of individuals who choose to spend their leisure time outdoors engaging in outdoor recreational activities in natural spaces. During the pandemic, people are more likely to maintain their physical and mental health if they spend time outdoors in natural open spaces [4,5]. Consequently, it is crucial to consider whether the quality of recreational facilities can encourage the public to stay. High-quality recreation spaces must have good natural environment quality (NEQ) and support capabilities for recreation services (RS).

Apart from providing a place for people to relax and play, urban waterfront space also serves ecological functions [2], including climate regulation [6], air purification [7], rainwater storage [8], soil conservation, biological migration support [9], and biodiversity maintenance, among others. Urban waterfronts, as an ecological base and potential recreational space, can achieve multiple objectives and provide benefits in ecological protection [10], social and economic development [11], landscape enhancement, and public welfare [12]. However, recreational services must be coordinated with ecological functions to achieve this objective.

Numerous studies on waterfront space and residents’ perceptions of urban parks, including aesthetic, cultural, and safety studies, have been conducted [13]. To facilitate sustainable development and health in urban areas, researchers have paid more attention to the needs of the elderly, children, and the homeless [14,15,16]. In China, most waterfront research focuses on landscape design, landscape evaluation, sponge cities, and others [17]. When discussing parks and residents’ well-being, the focus is on dweller satisfaction, accessibility, public service facilities, and the balance between supply and demand [18]. Few studies examine the effect of natural ecological factors on the vitality of waterfront areas. The impact of the coupled coordination of natural ecological and social factors on the vitality of waterfront spaces has rarely been measured, but some scholars have used coordination coherence by some scholars to measure the relationship between urbanization and ecosystems [19,20].

Several factors contribute to the vitality of waterfront spaces, including their natural environment, historical and cultural background, spatial characteristics, popularity, and ability to represent the image of a city [21]. Existing studies use traditional methods, such as questionnaires, in-depth interviews, and field surveys, to quantify the vitality of waterfront space [16,22]. Typically, an analytic hierarchy process (AHP) and accessibility analysis are used to evaluate the function of waterfront space [23]. Moreover, there are numerous methods to analyze the space behavior characteristics of waterfront space through population heat map big data, mobile phone signaling data, and online social media big data, and then analyzing waterfront space environment preferences [24].

There are many well-designed urban waterfront spaces in China, but they attract few people and need more vitality. Two typical waterfront spaces were selected for this study, and the following questions were investigated by dividing the samples. (1) The connection between NEQ and RS. (2) How does residence appeal relate to the characteristics of the two waterfront spaces? Could coordination between the two boost spatial vitality? What landscape optimization strategies can be implemented in the context of developing ecological–recreational coupling to enhance spatial vitality and residency? It is necessary to determine whether the social behavior of the residents will conflict with or be constrained by the park’s design to determine whether the park’s design will facilitate the transition of people from behavioral activities to social interactions. By analyzing the characteristics of waterfront space and NEQ, social relations and social structures within the recreation process can be investigated.

Prior research has focused on the ecological or social effects of the site as independent studies, and there needs to be more research analyzing the crowd use of the space from the perspective of ecological and social coupling. To this end, we present the evaluation of RS and NEQ to measure the social recreational and natural ecological attributes of waterfront space, respectively, and we employed a coupled coordination model to assess the degree of social–ecological interaction and mutual influence in waterfront spaces. Using the degree of coupling and coordination between RS and NEQ and the vitality of waterfront space as independent variables, this study investigated the influence of waterfront space characteristics on waterfront vitality from the perspective of social–ecological coupling through linear and non-linear fitting and redundancy analysis. For waterfront space design and social behavior research, we propose environmental enhancement strategies based on varying degrees of coupling and coordination. Under the perspective of social–ecological composite, the influence of waterfront space characteristics on spatial vitality is investigated to provide a foundation for the multi-objective synergistic development of waterfront space.

## 2. Materials and Methods

### 2.1. Study Area

**Figure 1:**
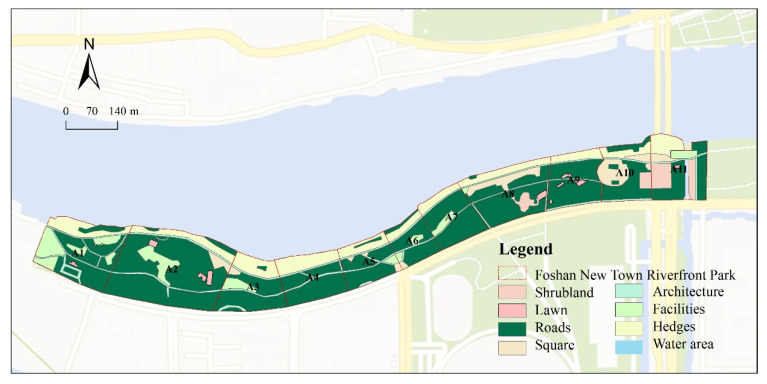
Landscape distribution map of New City Riverfront Park in Shunde District, Foshan City.

**Figure 2:**
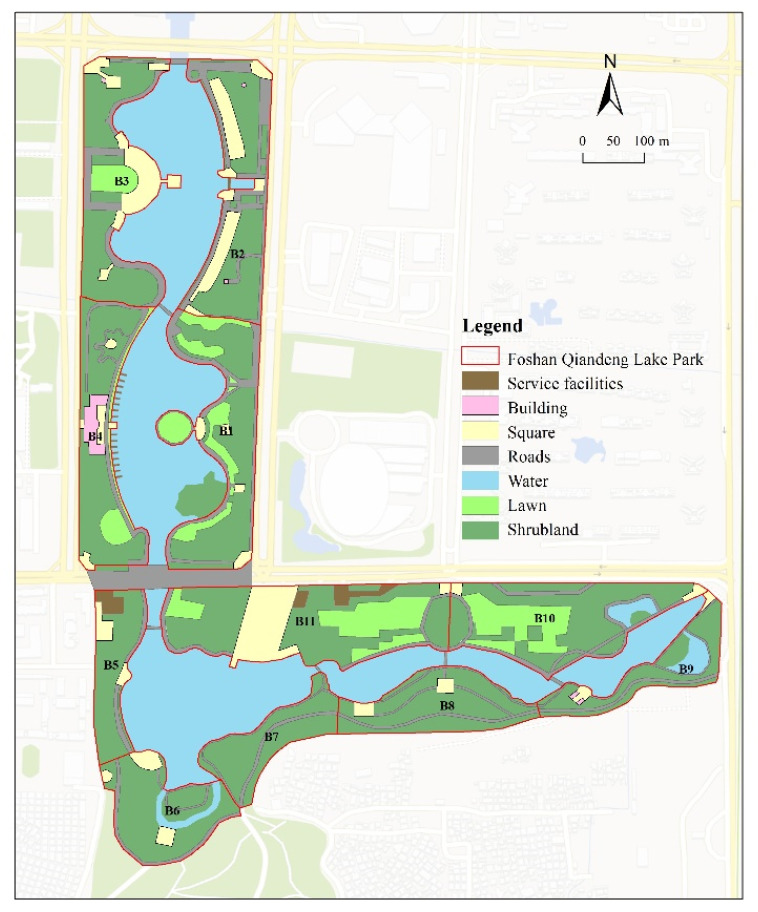
Landscape distribution map of Qiandeng Lake Park in Nanhai District, Foshan City.

This paper selected the New City Riverfront Park in Shunde District, Foshan, China (Figure 1), and the Qiandeng Lake Park in Nanhai District, Foshan, China (Figure 2), as sample sites. Both are located within the ecological space of Foshan City’s “Qianmu Park Ring” planning document. Foshan City published the “*Special Plan for the Construction of Natural Ecological Civilization in Foshan City*” in 2018, which proposed the construction of a multi-level life synergistic natural ecological pattern consisting of three screens, six wedges, two veins, two rings, and two networks. Given that the city of Foshan owns Qianmu Park Ring, a significant ecological space, the two riverfront spaces must be situated in an ecologically sensitive area. While NEQ plays a vital role in ensuring the ecological quality of the Qianmu Ring of urban parks in the city, the two sample sites are urban public spaces that must provide RS for their residents. Qiandeng Lake Park is a typical urban waterfront park with its narrow north–south water surface and connection to the Foshan waterway. New Town Riverfront Park was designed and constructed along the Tanzhou waterway and is a standard strip of waterfront space. The two sites are surrounded by residential land and are situated in densely populated regions with the activity of waterfront spaces, typical waterfront landscapes, and more extensive services. In this regard, it is appropriate to research the vitality factor of waterfront space quality and propose spatial optimization strategies to address this issue.

### 2.2. Study Contents

ENVI (The Environment for Visualizing Images) software, ArcGIS software, and Google Maps were used to interpret the images of New Town Riverfront Park and Qiandeng Lake Park in this paper [22]. ENVI is a comprehensive image-processing platform for remote sensing. We identified 22 research samples for studying the effects of waterfront space on recreational behavior based on the interpretation results. The samples were delineated to ensure that every sample had a water-friendly surface. The New Town Riverfront Park was separated into shrubs, lawns, water, roads, squares, buildings, facilities, hedges, and other spaces. In contrast, the Qiandeng Lake Park was separated into shrubs, lawns, water, roads, squares, buildings, facilities, hedges, and other spaces. The survey period was 1–3 October 2022 (National Day holiday), and the behavior notation method [25] was used to conduct the site survey. Image interpretation and site survey-based data were processed and analyzed using coupled coordination calculation, correlation analysis, and standardization.

### 2.3. Data and Methods

**Figure 3:**
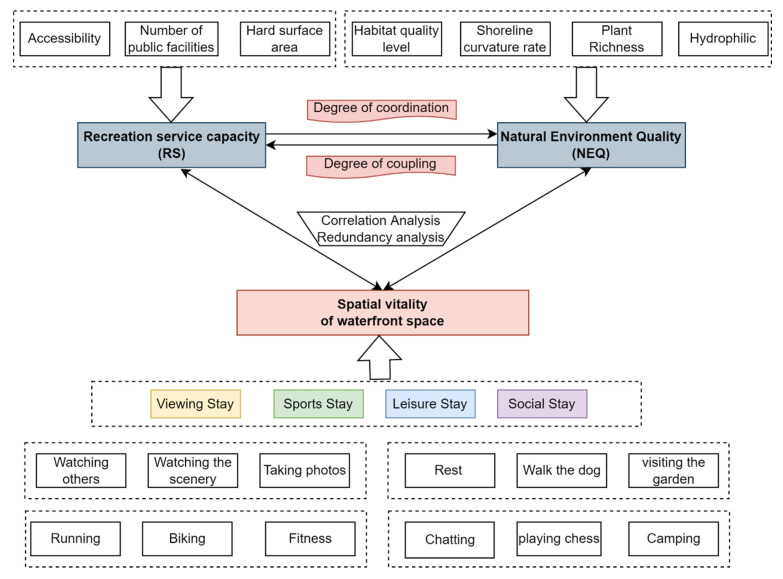
Indicator procedure diagram.

Combining the existing literature and the physical characteristics of the waterfront space [2,3,10], this paper selected NEQ and RS as the primary indicators to determine how the NEQ and RS of waterfront space influence recreational behavior and which factors influence waterfront space vitality (Table 1). The RS capacity quantifies the service function provided for recreation activities in a physical environment. Accessibility, public services, and the percentage of hard surface area were selected to explain RS variables based on existing research and the spatial environmental characteristics of waterfront spaces. Natural ecological function characteristics of waterfront space consist of habitat quality, shoreline curvature rate, plant richness, and hydrophilicity (Figure 3). The habitat quality module of the InVEST model was used to calculate indicators of biodiversity and ecological function.

The spatial vitality of waterfront space was measured using the behavioral notation method by recording the number of people staying in each sample and the activities they participated in. During the data collection period, the average temperature was 30 °C, and the weather ranged from sunny to cloudy. In order to collect data, the busiest times (9:00 to 12:00 and 16:00 to 19:00) were chosen. This paper used the coupled coordination model [20,21] to calculate the coupled coordination degree of NEQ and RS. The coupled coordination model determines the degree of interaction and mutual influence between the social and natural systems and reveals the law of development from uncoordinated to coordinated. Furthermore, the Pearson correlation analysis [26] between NEQ and RS was performed utilizing SPSS software, and a redundancy analysis model [27] was constructed utilizing Canoco 5 software (http://www.canoco5.com/) to examine the impact of NEQ and RS on the allure of waterfront space.

## 3. Results

### 3.1. Natural Environment Quality and Recreation Service of Waterfront Space

#### 3.1.1. Correlation Analysis of Natural Environment Quality and Recreation Service

**Figure 4:**
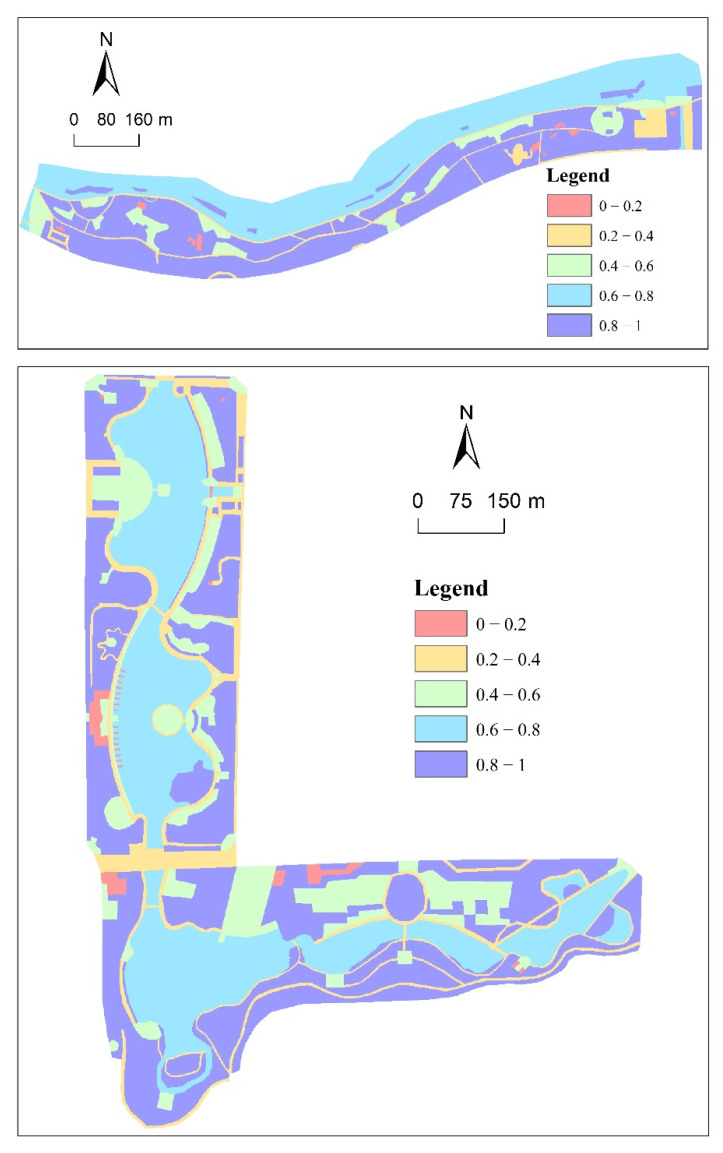
Habitat quality of Foshan New Town Riverside Park and Foshan Qiandeng Lake Park.

**Figure 5:**
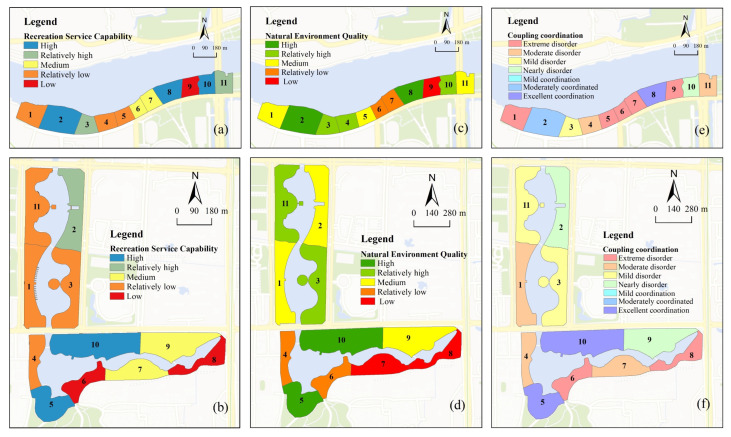
(**a**,**b**) Recreation service capacity. (**c**,**d**) Natural environment quality. (**e**,**f**) Coupling and coordination capacity.

Accessibility, the number of public facilities, and hard surface area are the three indicators that comprise RS. Four indicators make up the NEQ: habitat quality, shoreline curvature rate, plant richness, and hydrophilicity. InVEST software [28] was used to calculate the habitat quality level factor based on the four land-use types of squares, buildings, service facilities, and roads, which served as threat factors, according to the reference literature and field survey. Sensitivity coefficients and threat factors were assigned to calculate the habitat raster, and habitat quality was classified into five levels (Figure 4 and Figure 5). According to the statistics, samples A2, B11, and B10 had the highest levels of habitat quality; they were 48,427.97, 36,131.36, and 30,079.23, respectively. Samples A5, B5, and A6 had the lowest habitat quality levels; they were 13,193.18, 12,579.77, and 10,970.48, respectively. Sample A1 contained a high level of plant richness, whereas sample A9 contained the lowest level (Figure 4). The greatest plant richness was found in sample square A1, while the least was in sample square A9.

The Pearson correlation analysis and descriptive analysis of waterfront space characteristics using SPSS software demonstrated (Table 2 and Table 3) that accessibility has a significantly high positive correlation with the percentage of hard surface area (0.825) and plant diversity (0.790), indicating that people prefer to enter spaces with more hard surface areas and plants. Second, there was a strong positive correlation between habitat quality and the rate of shoreline curvature (0.729) and plant richness (0.585), indicating that spaces with curved shorelines and a greater variety of plant species have a higher level of biodiversity and a superior natural environment. According to the statistics, there is a strong positive correlation (0.761) between the two spatial characteristics of the waterfront.

#### 3.1.2. Analysis of Coupling Coordination Degree of Waterfront Space’s Spatial Characteristics

As shown in Table 4 and Figure 5, the RS and NEQ of waterfront space in Foshan City exhibited significant spatial differences due to the study samples’ distinct resource environments and development orientations. The highest capacity for RS (Figure 5a,b) was found in A2, A8, A10, B5, and B10, while the lowest capacity was found in A9, B6, and B8. This was because A2 and A8 have a high number of recreation facilities and A10 has a high percentage of hard surfaces, while A9 has a low hard surface area, and A9 has low accessibility. The quality of the natural environment was higher in A2, A8, B5, and B10 and lower in A6, A7, A11, B7, and B8 (Figure 5c,d). Overall, ecological–recreational coupling coordination was low, with 81.8% of sample sites dysfunctional and only four sample sites (A2, A8, B5, and B10) exhibiting ecological–recreational coupling coordination (Figure 5e,f). Overall, if the two sample sites are to better support the function of urban ecological corridors and serve public recreation, the RS and NEQ should be better balanced and developed in a coordinated manner. The potential for ecological–recreational coupling and coordinated development should be increased.

### 3.2. Resident Activities and Waterfront’s Spatial Vitality

#### 3.2.1. Resident Activity Analysis

The social behavior stay activities in Foshan City’s waterfront space can be classified into four groups: viewing stay, sports stay, leisure stay, and social stay. The viewing stay includes people watching, sightseeing, and photography (cell phones, drones); the sports stay includes running, cycling, and fitness (playing swords, shuttlecock, badminton, and basketball); the leisure stay includes resting (taking a cool break, dining, and drinking), walking dogs, and visiting the garden; and the social stay includes conversing, playing chess, and camping.

The two waterfront spaces differ in terms of the activities available during stays. In the Foshan New Town Riverfront Park, various activities are available. The composition was more balanced, with viewing (32.2%), leisure (26.3%), sports (24.2%), and social stays as the most frequent. The primary purpose of viewing activities is to provide an opportunity to observe the beautiful riverfront ecological environment and enjoy the scenery. The park is home to 323 kinds of plants and over 60 species of birds throughout the year. In addition, there are flowering areas such as flower seas, flower fields, flower belts, flower forests, and flower borders. Meanwhile, the riverside park features a playground, musical fountain, five-a-side soccer field, basketball court, skateboard field, fitness stage, and jogging track, among other sporting amenities. Viewing stays accounted for 41.2% of all stays in Qiandeng Lake Park, while leisure stays accounted for 27.1%. People choose to spend time in the waterfront area because of its aesthetic appeal and points of appreciation. Space B is distinguished by its large water fountain installation, shoreline wetland with various plant configurations, and comprehensive park services and facilities.

When comparing the time distribution of stay activities, the most noticeable difference between Foshan New Town Riverside Park and Qiandeng Lake Park was that Qiandeng Lake Park had more people staying at night. In the summer, the sun is intense during the day, and there is little shade on the park’s waterfront, causing the number of people to peak at 19:00. The park’s lighting and fountain configurations are more distinctive, attracting a large number of visitors. In contrast, Foshan New Town Riverfront Park lacks adequate lighting at night, it has poor visibility, the landscape on the opposite side of the riverfront is unattractive, and there are no food service facilities. As a result, the number of visitors is very different during the day and at night.

**Figure 6:**
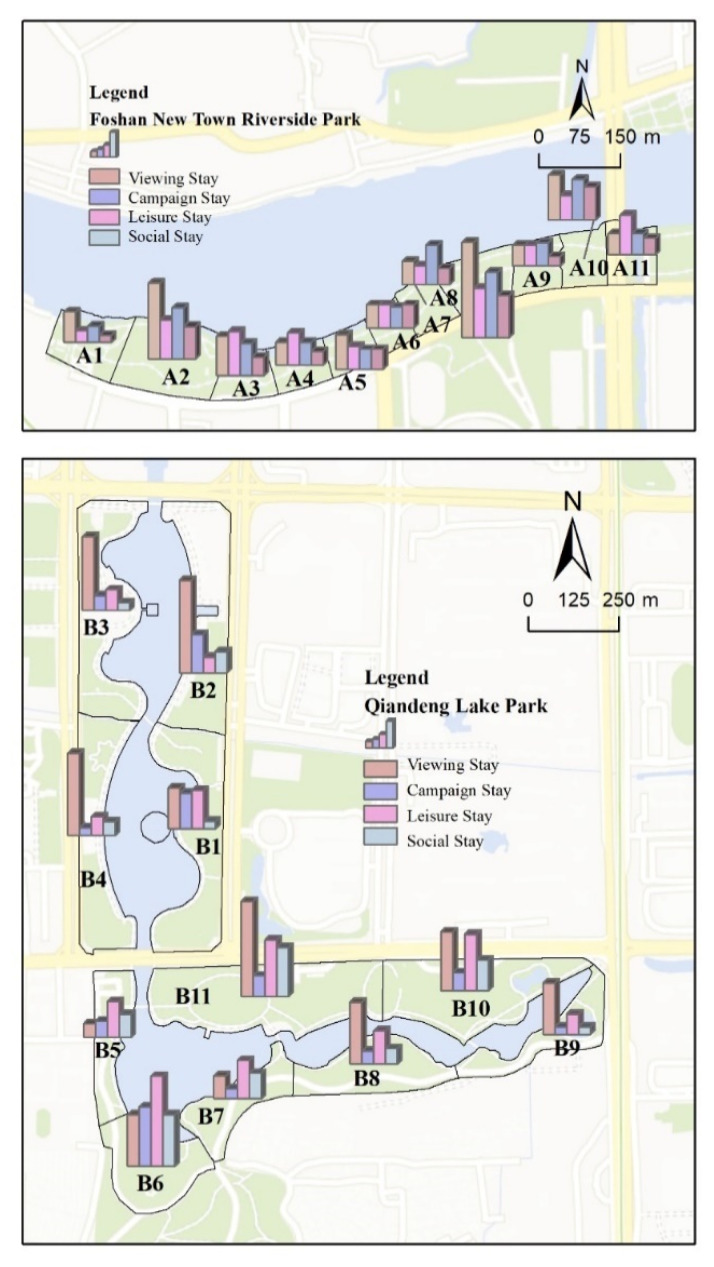
Vitality values of the different activity types in the waterfront spaces.

In order to assess the level of attraction of various waterfront spaces, it is necessary to observe the number of people staying in each space. A8 (18.7%) and A2 (14.8%) had the highest stay percentage across the entire A space sample section. Sample A8 includes a larger square to create a spatial enclosure, resting seats, and a lawn for visitors to sit on to feel safe and comfortable during their stay. It helps visitors feel protected and offers privacy for viewing and leisure activities. The hedge along the shoreline of sample A2 is shorter than that of sample A1; consequently, the view of the river becomes more expansive, enticing people to stay and take photos of the scenery. We discovered that B6 (16.09%), B2 (10.73%), and B11 (14.12%) had significantly higher stay rates than any other B space. Sample B6 provides more leisure time due to its abundance of trees, which provide visitors with shady and cool resting areas during the summer. Sample B2 has an attractive promenade intended to entice visitors to congregate and stay; sample B11, which serves as the park’s main entrance, has a large open square, a smaller square with shade trees, and water spraying facilities to entice visitors to stay for longer periods. In general, there are significant differences between the types of stays in the samples (Figure 6), with viewing stays > leisure stays > sports stays > social stays in terms of the activities that occur during these stays. Sample squares A8, B6, and B11 had an overall higher level of attractiveness than sample squares B9, A9, and A1.

According to the Pearson correlation analysis of the total number of stay activities, there was a significant relationship between the number of various stay activities (Table 5). There was a significant correlation between people-watching and sightseeing (0.769), taking photos (0.710), and chatting (0.499); viewing and taking photos (0.884) and gardening (0.468); sports stays, leisure stays, and most of the various stay activities. This indicates that various behaviors will be performed concurrently in the space of the stay, as well as a variety of stay activities that are suitable for this space simultaneously. For instance, when a waterfront space has a high-quality natural environment, people enjoy various viewing activities, such as viewing and taking photos. In areas with excellent RS facilities, multiple sports activities, such as running, cycling, and fitness activities, can also occur simultaneously.

#### 3.2.2. The Relationship between Waterfront Spatial Characteristics and Stay Activities

As shown by the statistics (Table 6), there was a differential correlation between the RS and the NEQ and the viewing, leisure, sports, and social stay activities. During the viewing stay, there were significant correlations between people-watching behavior and recreation facilities (0.692), viewing behavior and accessibility (0.692), and photo behavior and shoreline curvature (0.732). People were more likely to observe behavior in spaces with recreation facilities and were more likely to observe behavior in spaces with high accessibility; spaces with a curved shoreline were more conducive to people enjoying the scenery and taking photos. In the sport stay, running and hydrophilicity (0.728) and fitness and accessibility (0.671) were highly significant, indicating that people who run prefer to stay at the shore for exercise because the space is more open and the air is fresher; however, fitness prefers highly accessible spaces. There was a significant positive correlation between rest and plant richness (0.749), dog walking and plant richness (0.645), gardening and accessibility (0.762), and plant richness (0.745) during leisure stays. In addition to demonstrating that behaviors are more likely to occur in environments with high accessibility and plant diversity, resting and dog walking were also more likely to occur in such settings. Chatting was highly associated with accessibility (0.836) and hard surface area share (0.814) among social stays; playing chess was highly associated with accessibility (0.621); and camping was highly associated with recreational facilities (0.403). Thus, social behaviors such as chess and chit-chat were more likely to occur in highly accessible and hard-surfaced areas. In contrast, camping was more prevalent in areas with a high concentration of recreational facilities.

The accessibility of a space determines a person’s behavior during his or her stay, as people are more likely to enter spaces with relatively good recreation facilities and abundant vegetation for recreational activities if they are easily accessible. Accessibility (0.905), public facilities (0.895), and plant richness (0.889) all contributed significantly to the vitality of spatial activity.

Positive correlations existed between RS and social retention (0.861), NEQ and viewing stay (0.675), and RS and NEQ coupling coordination degree and social stay (0.818). This study demonstrated that the quality of recreational facilities could increase social stays, whereas the quality of the natural environment can increase viewing stays. When the quality of the natural environment is high, people are more likely to stay viewing activities for longer durations. As a result of the combination of ecological and recreational activities, the waterfront space attracts a larger crowd than its influence alone would suggest. Overall, the correlation between both and total stay dynamics was strong and statistically significant, but the coupled coordination of RS and NEQ had a greater impact on total stay dynamics (Table 7).

### 3.3. Model Results

#### 3.3.1. Multiple Linear Regression

Multiple linear regression analysis was conducted using SPSS software to examine the linear relationship between waterfront spatial characteristics and stay activities. Utilizing both independent variables, we constructed a multiple linear regression model [29,30] using the waterfront spatial characteristics of RS and NEQ as independent variables and the total stay vitality as dependent variables. The regression coefficients, waterfront spatial characteristics, and total stay vitality are shown in the table below. According to the adjusted R-squared value, the model was both feasible and statistically significant, with an overall significance of *p* < 0.01, demonstrating the model’s feasibility and statistical significance (Table 8).

According to the regression results for stay attractiveness, several factors influenced the attractiveness of stay behavior: waterfront spatial characteristics of recreational facilities (b’ = 0.460), accessibility (b’ = 0.289), and shoreline curvature rate (b’ = 0.197). According to the significance results, these factors were statistically significant and positively correlated. In addition, the results indicated that recreational facilities, accessibility, and shoreline curvature are the factors that significantly impact the vitality of waterfront areas. In other words, the waterfront characteristics most likely to affect the public’s stay are the accessibility of the waterfront space, the condition of the recreational facilities, and the shoreline curvature. Therefore, the more accessible the waterfront space, the more abundant and diverse the recreational facilities, and the more curved the waterfront shoreline, the more willing people are to stay.

#### 3.3.2. Linear Fitting

**Figure 7:**
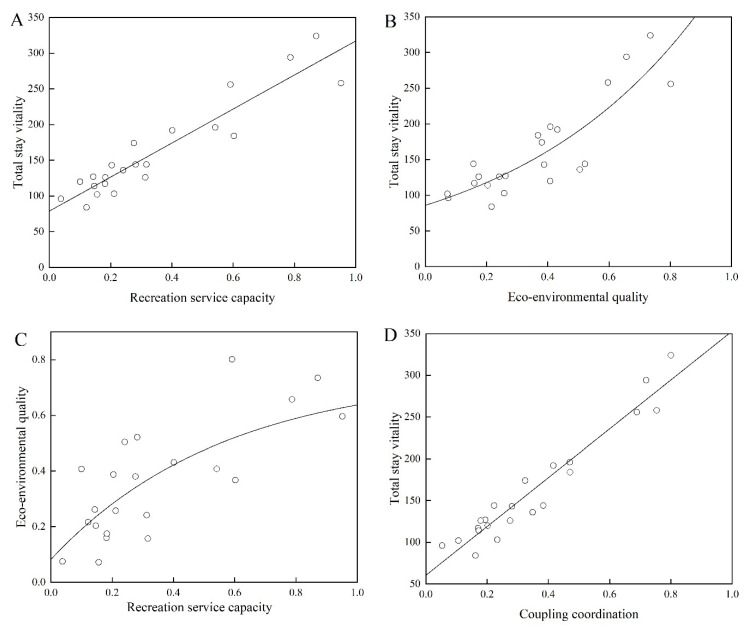
Results of fitting spatial characteristics of waterfront with spatial vitality values. (**A**) The llinear fitting between total stay vitality and RS; (**B**) The llinear fitting between total stay vitality and eco-environmental quality; (**C**) The llinear fitting between eco-environmental quality and RS; (**D**) The llinear fitting between total stay vitality and coupling coordination.

**Figure 8:**
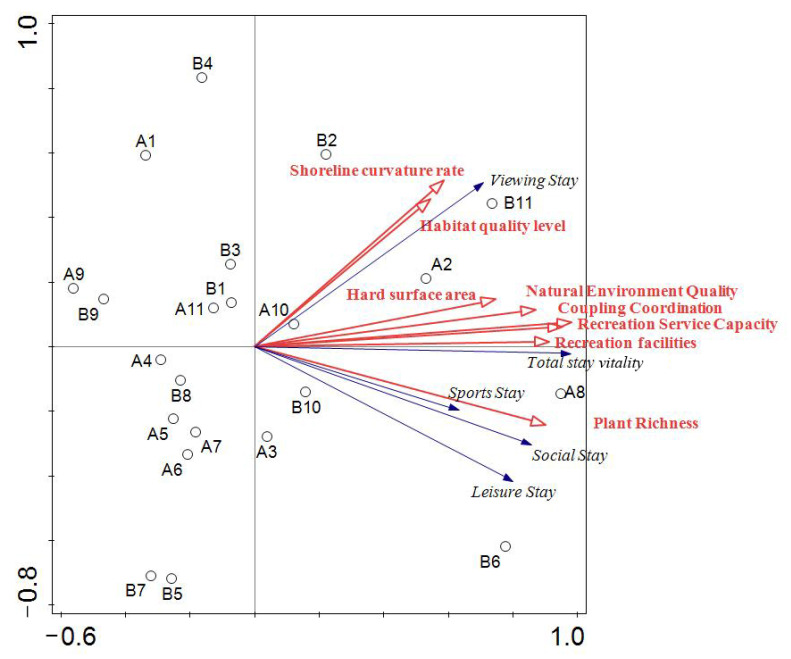
RDA biserial plots of spatial characteristic values and vitality values of the waterfront (*p* = 0.002 < 0.05).

Further linear fitting (Figure 7) was performed, and the simulation results demonstrated improved waterfront spatial characteristics and a higher total stay vitality according to the model’s level of explanation and significance. According to the fitting results, the RS, the degree of ecology–recreation coupling, and waterfront spatial vitality all exhibit a linear relationship. This indicates that the spatial vitality varies linearly with the degree of ecology–recreation coupling coordination and is, to some extent, related to those alterations. (1) Based on the curve fitting of NEQ and waterfront spatial vitality, it has been demonstrated that in a poor ecological environment, spatial vitality increases slowly as the environment is improved during the early stages of improvement and that the degree of spatial vitality increases significantly after ecological environment improvement (Figure 7B). (2) RS and overall stay behavior vitality demonstrate a clear linear relationship; as RS improves, the overall stay vitality of the waterfront space tends to rise with a slope of “238.24 ± 20.14.” However, coupled ecology–recreation coordination and overall stay behavior vitality demonstrate a clear linear relationship. Although the coupled ecology–recreation coordination and the overall stay dynamics also exhibited a linear relationship, the coupled coordination effect increased with a slope of “292.62 ± 18.25,” which was more effective than the effect of each variable separately (Figure 7A–D). (3) As depicted in Figure 8, the NEQ and RS curves were fitted to the space. While appropriate RS can help improve the spatial vitality of the space, excessive RS damages the NEQ, restricting the development of the natural environment and reducing spatial vitality.

#### 3.3.3. Redundancy Analysis

The fitting matrix correlation relationship of multivariate multiple linear regression was further demonstrated, and the redundancy analysis model (RDA) [31] of NEQ, RS, NEQ and RS coupling coordination, and spatial vitality characteristics was developed. Red arrows represent waterfront spatial characteristics, blue arrows represent stay attractiveness, and black circles represent samples. There was a positive and negative correlation between waterfront spatial features and stay attractiveness in the quadrant where the arrow is located. The ray length indicates how strongly correlated a certain waterfront spatial feature factor was with stay attractiveness; the longer the ray extends, the stronger the correlation, and vice versa. The graph shows that the NEQ, ecology–recreation coupling degree, and RS (hard surface area, recreation facilities) are all in the same quadrant. The ray extension is long, and the angle is small, indicating a strong positive correlation between the factors. There was also a strong positive correlation between these spatial characteristics and the overall attractiveness of the waterfront. A direct relationship existed within a specific range of values between waterfront spatial vitality and waterfront spatial characteristic values such as NEQ, ecology–recreation coupling degree, and RS. Moreover, the viewing stay strongly correlated with shoreline curvature and habitat quality in the first quadrant. In the fourth quadrant, plant richness strongly correlated with sports stay and social stay (Figure 8).

## 4. Conclusions and Discussion

### 4.1. Conclusions

(1) The vitality of waterfront spaces is primarily determined by RS capacity (accessibility, number of public facilities) and NEQ (plant richness, shoreline curvature). RS is essential for a social stay, while NEQ is more appealing for a viewing stay. There is a positive correlation between the degree of ecological–recreational coupling and social stay.

(2) At a low level of NEQ, waterfront spatial vitality increases gradually as the environment improves. NEQ and RS coupling coordination on waterfront spatial vitality is positively correlated; RS positively impacts spatial vitality. However, after reaching a certain level, RS uncontrolled growth will impede the development of the NEQ. Therefore, it is essential to recognize that, beyond a certain threshold, the unchecked growth of RS can slow the development of NEQ and even lead to a decline in spatial vitality.

(3) The relationship between ecology and recreation has a continuous positive effect on the spatial vitality of the region. However, individual waterfront spatial characteristics have less influence on the overall attractiveness of a place of residence than their combined development. Therefore, facing the development of dual social–ecological objectives, directing the synergistic growth of NEQ and RS is essential for the sustainable development and enhancement of urban waterfront space.

### 4.2. Strategies to Enhance Resident Attraction

Openness, continuity, and permeability are defining characteristics of the landscape design of waterfront spaces [32]. The waterfront space must be permeable to create more space between the vibrant downtown area and the natural environment, as well as open, allowing the water to provide a greater amount of viewing surface in the city park to accommodate the recreational preferences of those who prefer water activities.

#### 4.2.1. Enhance Recreation Service Facilities and Accessibility in Order to Expand Recreation Service Capacity

The quantity of RS available to the public is one of the most critical factors contributing to the vitality of waterfront spaces. People can stay in the waterfront space for various reasons, including viewing, leisure, sports, and socializing, provided that the waterfront space is accessible, and depending on the ratio of hard surface area and the number of RS facilities present. First, we must not only rationalize the distance between the park’s entrance and exit but also improve the internal articulation of the landscape design to attract visitors and make them intuitively want to stay in the space [33]. The hedge at the park’s entrance should not be too large or too long, as this will directly impede the movement of visitors; however, the square itself can be too large for people to linger and they will feel unsafe. According to Yoshinobu Ashihara’s “Aesthetics of the Street,” a plaza should have a sense of enclosure. However, its connection to the street should be permeable so that people can enter the space and enjoy the activities therein with joy. Secondly, to create a desirable waterfront space, there must be sufficient seating, bulletin boards, signs, tables, street lighting, toilets, statues, and trash cans, as well as the proper placement of these amenities. This study found that in Sample A, the lack of public service support for street lighting and catering in this waterfront space resulted in fewer evening visitors compared to daytime hours.

#### 4.2.2. Enhance the Natural Environment, Optimize NEQ, and Increase Residence Attraction

**Figure 9:**
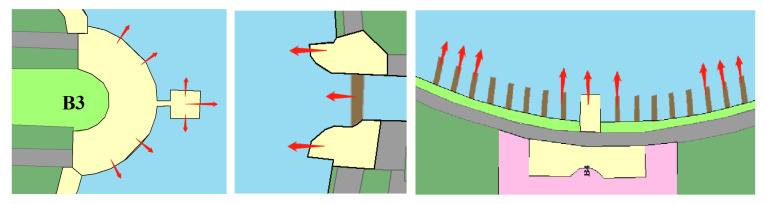
Convex shoreline, horizontal + convex shoreline, and concave + convex shoreline.

Habitat quality level is one of the effective indicators to measure the habitat quality and degradation level of the landscape based on land cover. Creating and maintaining natural habitats is one of the most critical methods for attracting visitors and increasing their propensity to stay in the park. A complete ecological space is conducive to adjusting the microclimate of the waterfront space on the one hand, and it enhances people’s aesthetic perceptions on the other hand. The treatment of the shoreline is closely related to the waterfront’s viability and making it one of the most desirable places for tourists to stay. As a means of enhancing the accessibility of the transitional shoreline, the convex treatment can lengthen the shoreline. In contrast, the concave treatment can attract people’s attention and encourage them to enter the waterfront area [34]. Foshan Qiandeng Lake Park, for instance, can diversify its shoreline treatment, and each shoreline can attract visitors to stay (Figure 9).

#### 4.2.3. Promote the Ecological–Recreational Coupling of Waterfront Space

**Figure 10:**
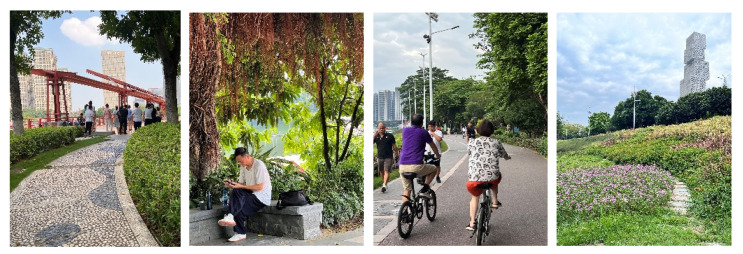
Social stay in the square, leisure stay in the shade, sport stay on the trail, and viewing stay on the flower trail.

The waterfront space with ecological–recreational coupling coordination can be roughly divided into three levels: 1–4 for dysfunctional coupling coordination, 5 for close coupling coordination, and 6–10 for good coupling coordination [35]. As a space with a relatively high occupancy rate, the waterfront space with a high stay rate typically has a relatively ideal ecosystem and recreational system. A viewing stay in the flower path, for example, must be characterized by layers and varieties of rich plants and continuous and complete trails. In contrast, a social stay in a square requires open, paved surfaces and road accessibility [36]. The leisure stays in the shade depend on good tall shrubs and seating facilities being combined to facilitate rest after exercise. In contrast, the sports stay on a trail considers the variety of paving and street light configurations. It is essential to emphasize that the NEQ and RS of the waterfront space need to be coupled and coordinated, which contributes to the space’s vitality. Consequently, ecological–recreational multi-objective development and composite site design [37] are considered in the design of waterfront space and are essential for creating a dynamic waterfront space and diversifying social behavior activities there (Figure 10).

### 4.3. Limitations and Future Directions

This paper has apparent advantages and significance for comprehensively studying the ecological and social vitality of waterfront areas. Nonetheless, a more comprehensive study can include additional objectives, such as cultural and social well-being indicators; selecting the ecological and social indicators and the weighting of indicators can also be more scientific and diverse. This framework can be applied to urban waterfront spaces, but verification is still necessary. Our evaluation framework can serve as a benchmark and innovation for assessing the rationality of waterfront space design and the vitality of individuals. In the future, the development of waterfront spaces and people’s behavior are likely to become more diverse, necessitating an update to the content and research methods to reflect the characteristics of the sites.

## Figures and Tables

**Table 1:** Calculation and data acquisition methods for the spatial characteristic variables of green space.

Spatial Variable Characteristics	Indicator Layer	Calculation Formula	Acquisition Method
Recreation service capacity (RS) R = (RS a + RS f + RS h)/3	Accessibility (RS a)	RSa = length of sample entrance/sample circumference	Remote sensing data, Baidu map, field research
	Number of public facilities (RS f)	RSf = number of public recreation, service, and management facilities	Field research
	Percentage of hard surface area (RS h)	RSh = hard surface/sample area	Remote sensing data
Natural environmental quality (NEQ) E = (NEQ h + NEQ c + NEQ p + NEQ d)/4	Habitat quality level (NEQ h)	InVEST model habitat quality and ArcGIS calculation	Park landscape (land use) type classification
	Shoreline curvature (NEQ c)	NEQ c = L/L’ shoreline/total shoreline	Field research, ArcGIS calculation
	Plant richness (NEQ p)	NEQ p = number of plant species	Site research
	Hydrophilicity (height difference/distance) (NEQ d)	NEQ d = distance from water	Field research measurement
Coupling coordination degree (D) D = $\sqrt{C\times T}$	Coupling degree (C)	= $2\frac{\sqrt{R\times \;E}}{R+E}$	-
	Coordination degree (T)	T = a × RS + b × NEQ (a = 0.5, b = 0.5)	-

Note: The secondary classification 0 needs to be standardized to approximate the coupling coordination analysis.

**Table 2:** Descriptive statistics of waterfront characteristics.

Indicator Layer		Numbers	Minimum	Maximum	Average	Standard Deviation
RS	RS a	22	0.03	1.00	0.45	0.24
	RS f	22	0.00	1.00	0.33	0.30
	RS h	22	0.00	1.00	0.36	0.30
NEQ	NEQ h	22	0.00	1.00	0.27	0.24
	NEQ c	22	0.00	1.00	0.40	0.31
	NEQ p	22	0.00	1.00	0.39	0.28
	NEQ d	22	0.00	1.00	0.40	0.33

Note: Data were standardized prior to descriptive statistical analysis.

**Table 3:** Correlation analysis between the spatial characteristics of the waterfront.

Criteria Layer		RS			NEQ			
Indicator Layer		RS a	RS f	RS h	NEQ h	NEQ c	NEQ p	NEQ d
RS	RS a	1						
	RS f	0.769 **	1					
	RS h	0.825 **	0.567 **	1				
NEQ	NEQ h	0.452 *	0.571 **	0.294	1			
	NEQ c	0.527 *	0.394	0.474 *	0.729 **	1		
	NEQ p	0.790 **	0.823 **	0.581 **	0.585 **	0.569 **	1	
	NEQ d	0.391	0.338	0.298	−0.116	0.021	0.381	1

* Significant correlation at the 0.05 level (two-tailed). ** At the 0.01 level (two-tailed), the correlation is significant.

**Table 4:** Coupling coordination level.

D-Value Range	Coherence Level	Coupling Coordination Degree	Sample
[0.0–0.2)	Levels 1–2	Extreme disorder	A1, A5, A6, A7, A9, B6, B8
[0.2–0.3)	Level 3	Moderate disorder	A4, A11, B1, B4, B7
[0.3–0.4)	Level 4	Mild disorder	A3, B3, B11
[0.4–0.5)	Level 5	Nearly coordinated	A10, B2, B9
[0.5–0.6)	Level 6	Mild coordination	-
[0.6–0.7)	Level 7	Moderately coordinated	A2
[0.7–1.0)	Levels 8–10	Excellent coordination	A8, B5, B10

**Table 5:** Correlation analysis of total stay activity.

Guideline Level		Viewing Stay			Sports Stay			Leisure Stay			Social Stay		
Indicator Layer		Watching people	Viewing	Taking photos	Running	Biking	Fitness	Rest	Dog walking	Garden	Chatting	Chess	Camping
Viewing Stay	Watching people	1											
	Viewing	0.769 **	1										
	Taking photos	0.710 **	0.884 **	1									
Sports Stay	Running	−0.056	0.075	0.052	1								
	Biking	0.100	−0.100	−0.027	0.519 *	1							
	Fitness	0.218	0.370	0.329	0.483 *	0.276	1						
Leisure Stay	Rest	0.132	0.171	−0.019	0.657 **	0.056	0.411	1					
	Dog walking	0.472 *	0.284	0.294	0.463 *	0.640 **	0.348	0.361	1				
	Garden	0.246	0.468 *	0.276	0.544 **	−0.213	0.335	0.786 **	0.210	1			
Social Stay	Chatting	0.499 *	0.432 *	0.261	0.389	−0.033	0.538 **	0.673 **	0.376	0.617 **	1		
	Chess	0.060	0.326	0.147	0.314	−0.444 *	0.341	0.700 **	−0.077	0.717 **	0.591 **	1	
	Camping	0.388	0.124	0.164	0.408	0.702 **	0.002	0.244	0.649 **	−0.009	0.139	−0.343	1

* Significant correlation at the 0.05 level (two-tailed). ** At 0.01 level (two-tailed), the correlation is significant.

**Table 6:** Correlation analysis of waterfront spatial characteristics and total resident activities.

Criteria Layer		RS			NEQ			
Indicator Layer		RS a	RS f	RS h	NEQ h	NEQ c	NEQ p	NEQ d
Viewing Stay	Watching people	0.578 **	0.671 **	0.643 **	0.523 *	0.493 *	0.444 *	0.153
	Viewing	0.692 **	0.685 **	0.589 **	0.621 **	0.609 **	0.581 **	0.109
	Taking photos	0.574 **	0.529 *	0.509 *	0.605 **	0.732 **	0.437 *	0.147
Sports Stay	Running	0.492 *	0.450 *	0.214	0.049	0.157	0.613 **	0.728 **
	Biking	−0.035	0.204	−0.075	−0.015	0.040	0.158	0.400
	Fitness	0.671 **	0.509 *	0.578 **	0.279	0.484 *	0.537 **	0.286
Leisure Stay	Rest	0.621 **	0.633 **	0.442 *	0.141	0.101	0.749 **	0.407
	Dog walking	0.369	0.613 **	0.345	0.534 *	0.413	0.645 **	0.320
	Garden	0.762 **	0.612 **	0.508 *	0.296	0.293	0.745 **	0.287
Social Stay	Chatting	0.836 **	0.714 **	0.814 **	0.166	0.193	0.677 **	0.419
	Chess	0.621 **	0.491 *	0.409	0.280	0.130	0.546 **	0.208
	Camping	0.104	0.403	0.104	0.071	0.133	0.355	0.300
Total Stay Vitality	-	0.905 **	0.895 **	0.729 **	0.522 *	0.566 **	0.889 **	0.473 *

* Significant correlation at the 0.05 level (two-tailed). ** At 0.01 level (two-tailed), the correlation is significant.

**Table 7:** Correlation analysis of waterfront spatial characteristics and total stay activities.

Variables	Viewing Stay	Sports Stay	Leisure Stay	Social Stay	Total Stay Vitality
RS	0.711 **	0.520 *	0.749 **	0.861 **	0.935 **
NEQ	0.675 **	0.600 **	0.658 **	0.627 **	0.853 **
RS and NEQ Coupling Coordination Degree	0.736 **	0.595 **	0.766 **	0.818 **	0.963 **

** Significant correlation at the 0.01 level (two-tailed). * At the 0.05 level (two-tailed), the correlation is significant.

**Table 8:** Table of regression model coefficients for spatial characteristics of waterfront and total stayvitality.

Independent Variables		Total Stay Vitality				
		Non-Standardized b	Standardized b’	t	Significance	VIF
	(Constant)	20.165	-	1.569	0.139	-
RS	RS a	0.286	0.289	2.069	0.057	6.500
	RS f	4.806	0.460	3.851	0.002	4.741
	RS h	25.440	0.370	0.359	0.725	3.524
NEQ	NEQ h	−0.001	−0.114	−1.086	0.296	3.685
	NEQ c	421.322	0.197	1.999	0.065	3.224
	NEQ p	0.763	0.174	1.450	0.169	4.767
	NEQ d	22.488	0.110	1.620	0.128	1.536

## Data Availability

The datasets used or analyzed during the current study are available from the corresponding author on reasonable request.
